# Clinical-level screening of sleep apnea syndrome with single-lead ECG alone is achievable using machine learning with appropriate time windows

**DOI:** 10.1007/s11325-025-03316-0

**Published:** 2025-04-11

**Authors:** Takahiro Yamane, Masanori Fujii, Mizuki Morita

**Affiliations:** 1 Department of Biomedical Informatics, Graduate School of Interdisciplinary Science and Engineering in Health Systems, Okayama University, Okayama, Japan; 2 Department of Geriatric Medicine, Faculty of Medicine, Dentistry and Pharmaceutical Sciences, Okayama University, Okayama, Japan; 3 Department of Allergy and Respiratory Medicine, Okayama University Hospital, Okayama, Japan; 4 Faculty of Health Sciences, Okayama University Medical School, Okayama, Japan

**Keywords:** Disease screening, Sleep apnea syndrome (SAS), Single-lead ECG, Artificial intelligence, Machine learning

## Abstract

**Purpose:**

To establish a simple and noninvasive screening test for sleep apnea (SA) that imposes less burden on potential patients. The specific objective of this study was to verify the effectiveness of past and future single-lead electrocardiogram (ECG) data from SA occurrence sites in improving the estimation accuracy of SA and sleep apnea syndrome (SAS) using machine learning.

**Methods:**

The Apnea-ECG dataset comprising 70 ECG recordings was used to construct various machine-learning models. The time window size was adjusted based on the accuracy of SA detection, and the performance of SA detection and SAS diagnosis (apnea‒hypopnea index ≥ 5 was considered SAS) was compared.

**Results:**

Using ECG data from a few minutes before and after the occurrence of SAs improved the estimation accuracy of SA and SAS in all machine learning models. The optimal range of the time window and achieved accuracy for SAS varied by model; however, the sensitivity ranged from 95.7 to 100%, and the specificity ranged from 91.7 to 100%.

**Conclusions:**

ECG data from a few minutes before and after SA occurrence were effective in SA detection and SAS diagnosis, confirming that SA is a continuous phenomenon and that SA affects heart function over a few minutes before and after SA occurrence. Screening tests for SAS, using data obtained from single-lead ECGs with appropriate past and future time windows, should be performed with clinical-level accuracy.

## Introduction

Sleep apnea syndrome (SAS) is a disorder in which breathing frequently ceases or decreases during sleep. Symptoms include snoring during sleep, extremely heavy daytime sleepiness, fatigue, and difficulty concentrating. SAS significantly impacts both the cardiovascular and endocrine systems and is closely linked to several lifestyle-related diseases, such as hypertension, heart failure, diabetes, and cerebrovascular disease [1]. SAS can be classified into three types: central sleep apnea (CSA), obstructive sleep apnea (OSA), and a combination of these two types. In CSA, breathing during sleep is disturbed by an abnormality in the respiratory center located in the medulla oblongata of the brain. In OSA, the pharynx is obstructed, and air cannot pass through [2]. The number of potential patients with OSA has been estimated to be more than that of patients treated with continuous positive airway pressure (CPAP), the standard treatment for moderate to severe OSA, regardless of the country. In Japan, for example, the former is estimated to be 22 million [3], while the latter is > 400,000 [4]. In France, the former is approximately 24 million [3], and the latter is approximately 830,000 [5]. These data indicate that the number of patients with OSA who are not properly diagnosed and benefit from treatment is very small.

SAS is diagnosed using polysomnography (PSG), which measures electroencephalogram (EEG), eye movements, electrocardiogram (ECG), electromyogram (EMG), and oxygen saturation at night. SAS is diagnosed when symptoms such as daytime sleepiness are present and when PSG shows five or more apneas/hypopneas per hour. Apnea is defined as cessation of breathing for more than 10 s, while hypopnea is defined as breathing that has not stopped completely but is less than half the airflow [6]. PSG is the gold standard for diagnosing SAS; however, there are challenges. For example, patients must wear many devices and sleep overnight in the hospital, which is a heavy burden on the patient. It is also expensive to implement, and few hospitals have specialists. These difficulties have led to increased hospital waiting times and reduced patient diagnosis rates. Thus, developing a simple and noninvasive method for automatically detecting sleep apnea (SA) at home by utilizing a reduced number of lead signals is essential.

Research has been conducted on methods for automatically detecting SA from PSG data and other sources. The acquisition of PSG data is not easy, and the number of parameters is large; therefore, the challenge is to reduce the number of parameters while maintaining detection accuracy. For example, by using only ECG data, one can estimate whether a certain minute (epoch) is an SA based on the interval and amplitude of the R-wave. The accuracy of SA detection can be improved by using ECG data from the previous 5 min before the epoch of SA occurrence [7] or by using data from 1 min before and after the epoch of SA occurrence [8, 9]. As SA is a continuous phenomenon [10] and has an influence on the ECG waveform further ahead [11], the accuracy may be further improved by using not only past but also future data. However, the effect of the combined use of past and future data, as well as the appropriate data range, has not yet been clarified.

The long-term objective of this study was to screen potential patients with SAS using a simple and noninvasive method, leading to increased diagnosis rates and appropriate treatment. The short-term objective was to confirm the usefulness of both past and future data for detecting SA and diagnosing SAS with machine learning using ECG data alone. We hypothesized that using past and future ECG data in a certain range would improve the SA detection accuracy and, thus, SAS diagnosis because SA occurs uninterruptedly, and ECG waveforms would be affected around the point of SA occurrence.

## Methods

### Dataset

The Apnea-ECG database from PhysioNet [12] was used to build and test the sleep apnea detection method. This dataset comprised a released and withheld set. Each set contained ECG recordings from 35 individuals, collected overnight. The sampling rate was 100 Hz, and the resolution was 16-bit. An expert marked an annotation on each 1-min segment of the ECG recordings. Annotation indicates the presence of apnea or hypopnea in the segment. There were 17,045 1-min segments in the released set and 17,268 1-min segments in the withheld set.

### Preprocessing

The ECG signal was preprocessed to obtain the RR intervals and R-peak amplitudes, as described previously [7]. Briefly, a finite impulse response (FIR) bandpass filter was used to remove noise from the original ECG signal. The cutoff frequencies were 3 and 45 Hz. The Hamilton algorithm was used to detect the R peaks [13]. A median filter was used to eliminate physiologically uninterpretable points in the generated RR intervals [14].

### Feature extraction, RR intervals features, and R-peak amplitude features

Twelve machine learning features were extracted from the RR intervals and six features from the R-peak amplitudes, as previously described [7]. The RR interval features comprised six time-domain and six frequency-domain features. The six time-domain features were MRR (mean of RR intervals), MHR (mean of heart rate), RMSSD (root mean square of differences between adjacent RR intervals), SDNN (standard deviation of RR intervals), NN50 (number of adjacent RR intervals exceeding 50 ms), and pNN50 (NN50 divided by the number of RR intervals) [15]. The six frequency-domain features were VLF (very low frequency, 0–0.04 Hz), LF (low frequency, 0.04–0.15 Hz), HF (high frequency, 0.15–0.4 Hz), LF/HF, LF/(LF + HF), and HF/(LF + HF), which were extracted from the power of each frequency component calculated using the Welch method with FFT (s, *N* = 256) [16]. The R-peak amplitude features comprised six frequency-domain features: VLF, LF, HF, LF/HF, LF/(LF/HF), and HF/(LF/HF). After feature extraction, the features were normalized to annul their distribution differences.

### Machine learning classifiers

Five frequently used machine learning classifiers were used to detect the SA: multilayer perceptron (MLP), support vector machine (SVM), random forest (RF), extreme gradient boosting (XGBoost), and logistic regression (LR). An MLP is an artificial neural network comprising three or more layers: an input layer, hidden layer(s), and an output layer. MLP can avoid the influence of data distribution on performance [17]. SVM is a supervised learning algorithm used for two-class classification. It creates a decision boundary between two classes to predict labels from one or more feature vectors [18]. RF is a combination of multiple decision trees that are dependent on the values of a random vector sampled independently. The class is determined by counting the voted numbers given by different decision trees [19]. XGBoost is a gradient tree boosting that makes a prediction by summing predictions from each decision tree [20]. LR is a regression model that predicts the probability of event occurrence [21].

### Hyperparameters

Some hyperparameters were altered from the default settings based on the results of hyperparameter tuning with optuna [22]. The hyperparameters “hidden_layer_sizes,” “alpha,” and “max_iter” used in MLP were (number of features × 2 + 1), 1, and 1000, respectively. Those “C” and “tol” used in SVM were 9.9 and 5 × 10^− 4^. Those “n_estimators,” “max_depth,” “max_features,” and “random_state” used in RF were 148, 185, 15, and 42, respectively. Those “eta,” “num_round,” “early_stopping_rounds,” “max_depth,” “min_child_weight,” “gamma,” “colsample_bytree,” and “subsample” used in XGBoost were 0.1, 10,000, 50, 9, 0.121, 6.45 × 10^− 7^, 0.876, and 0.872, respectively. The “random_state” used in LR was 42.

### Future and past time window

**Fig. 1:**
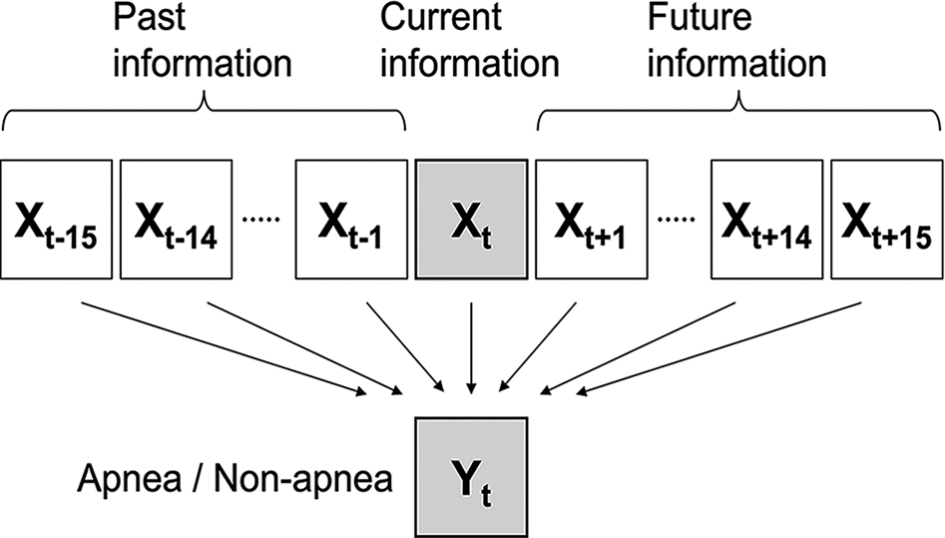
Example of time window scheme. The left part represents past information, the middle part represents current information, and the right part represents future information. The time window size was 15 min for both the future and past in this example

SA may last longer than 1 min and is time-dependent [8, 10]. Therefore, previous studies used ECG signal information prior to the point where SA occurred or adjacent 1-min information to detect it [7–9]. However, the effects of future information on SA detection have not been analyzed. Therefore, we used the future and/or past information in this study (Fig. 1). The future and past time window sizes were determined by comparing the accuracy of the SA detection results. To determine the optimal time window sizes, unless otherwise noted, the time window size was set to 1–15 min for both the future and past, and MLP was used as the classifier. Subsequently, the SA detection performance of each machine learning method was compared.

### Performance evaluation

The released dataset of 35 individuals from the Apnea-ECG database was divided into a training set (*n* = 28) and a validation set (*n* = 7), maintaining a 4:1 ratio. This split was used for group 5-fold cross-validation to assess SA detection performance by each machine learning classifier and determine the optimal time-window size. Subsequently, the entire 35-individual dataset used for the cross-validation was designated as the training group, whereas the withheld set of 35 individuals from the Apnea-ECG database was used as the test group. This group design allowed for evaluating each classifier’s performance with the optimized time-window size based on independent data.

Performance was evaluated using accuracy (ACC), sensitivity (SEN), specificity (SPE), precision (PRE), F1-score, the area under the receiver operating characteristic curve (AUC), and Pearson’s correlation coefficient. The equations for ACC, SEN, SPE, PRE, and F1-score are as follows:

$$\:\text{A}\text{c}\text{c}\text{u}\text{r}\text{a}\text{c}\text{y}\:=\:\frac{\text{T}\text{P}+\text{T}\text{N}}{\text{T}\text{P}+\text{T}\text{N}+\text{F}\text{P}+\text{F}\text{N}}$$

$$\:\text{S}\text{e}\text{n}\text{s}\text{i}\text{t}\text{i}\text{v}\text{i}\text{t}\text{y}/\text{R}\text{e}\text{c}\text{a}\text{l}\text{l}\:=\:\frac{\text{T}\text{P}}{\text{T}\text{P}+\:\text{F}\text{N}}$$

$$\:\text{S}\text{p}\text{e}\text{c}\text{i}\text{f}\text{i}\text{c}\text{i}\text{t}\text{y}\:=\:\frac{\text{T}\text{N}}{\text{T}\text{N}+\text{F}\text{P}}$$

$$\:\text{P}\text{r}\text{e}\text{c}\text{i}\text{s}\text{i}\text{o}\text{n}\:=\:\frac{\text{T}\text{P}}{\text{T}\text{P}+\:\text{F}\text{P}}$$

$$\:\text{F}\text{1}\text{-}\text{s}\text{c}\text{o}\text{r}\text{e}\hspace{0.17em}=\hspace{0.17em}2\:\times\:\:\frac{\text{P}\text{r}\text{e}\text{c}\text{i}\text{s}\text{i}\text{o}\text{n}\:\text{x}\:\text{R}\text{e}\text{c}\text{a}\text{l}\text{l}}{\text{P}\text{r}\text{e}\text{c}\text{i}\text{s}\text{i}\text{o}\text{n}+\text{R}\text{e}\text{c}\text{a}\text{l}\text{l}}$$

where TP, TN, FP, and FN denote the number of true samples classified as positive, true samples classified as negative, false samples classified as positive, and false samples classified as negative, respectively.

### Definition of the apnea‒hypopnea index (AHI) and SAS with AHI

The AHI was calculated from the number of SA detected using the following formula:

$$\:\text{A}\text{H}\text{I}\:=\:\frac{60}{\text{T}}\:\times\:\:\text{n}\text{u}\text{m}\:\text{o}\text{f}\:\text{S}\text{A}\:\text{s}\text{e}\text{g}\text{m}\text{e}\text{n}\text{t}\text{s}$$

where T is the recording time in minutes.

SAS was defined as AHI ≥ 5 in this study based on the practice guideline for OSA [23]. The diagnostic accuracy of SAS when the time window was expanded to the past and future was compared with that of the non-time window (without expanding the range of data used).

## Results

### Effect of time window size for per-segment SA detection in MLP

**Fig. 2:**
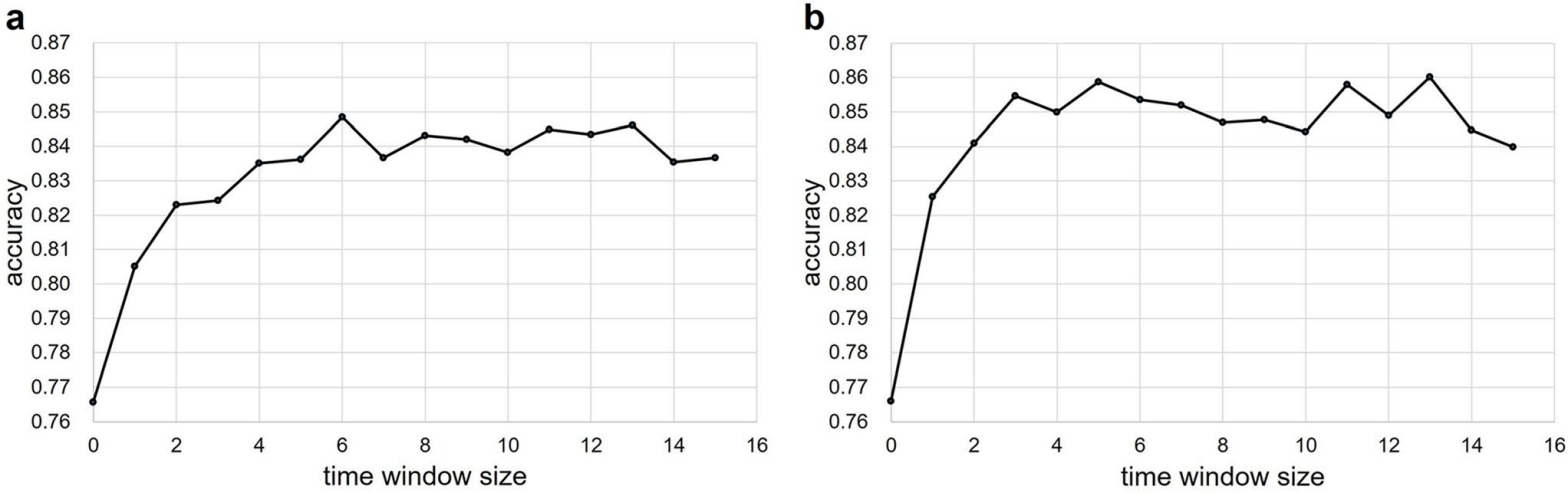
Five-fold cross-validation accuracy for different time windows on the released set. (**a**) Future data were input into MLP. The accuracy of (time window size = 6 min) was the highest. (**b**) Future and past data were input into MLP. The accuracy of (time window size = 13 min) was the highest

To examine the extent to which the occurrence of SA affects the ECG, the time window size was expanded in the future, and the change in the accuracy of SA detection was examined. As the time window size increased, accuracy increased, reaching a maximum at 6 min (Fig. 2a). The accuracy was saturated after 6 min, with a slight increase or decrease after 6 min. The accuracy increased the most when the time window size increased from 0 to 1 min.

To investigate how far in the future or in the past the occurrence of SA affects the ECG, the time window size was extended both in the future and in the past, and the change in the accuracy of SA detection was examined. The future and past were set to the same width. The accuracy increased as the time window size increased, reaching a maximum at 5 min, decreased, and then increased again, reaching a maximum at 13 min (Fig. 2b). The increase in accuracy was greatest when the time window size was extended from 0 min to 1 min.

### Effect of time window size for per-segment SA detection in multiple classifiers

The time window size was extended in the future by 1–15 min and in the past by 1–15 min. The accuracy of SA detection for each combination of extension widths was compared using a grid search. We used the five classifiers in our study to examine the relationship between the time window size and the accuracy of each classifier.

**Fig. 3:**
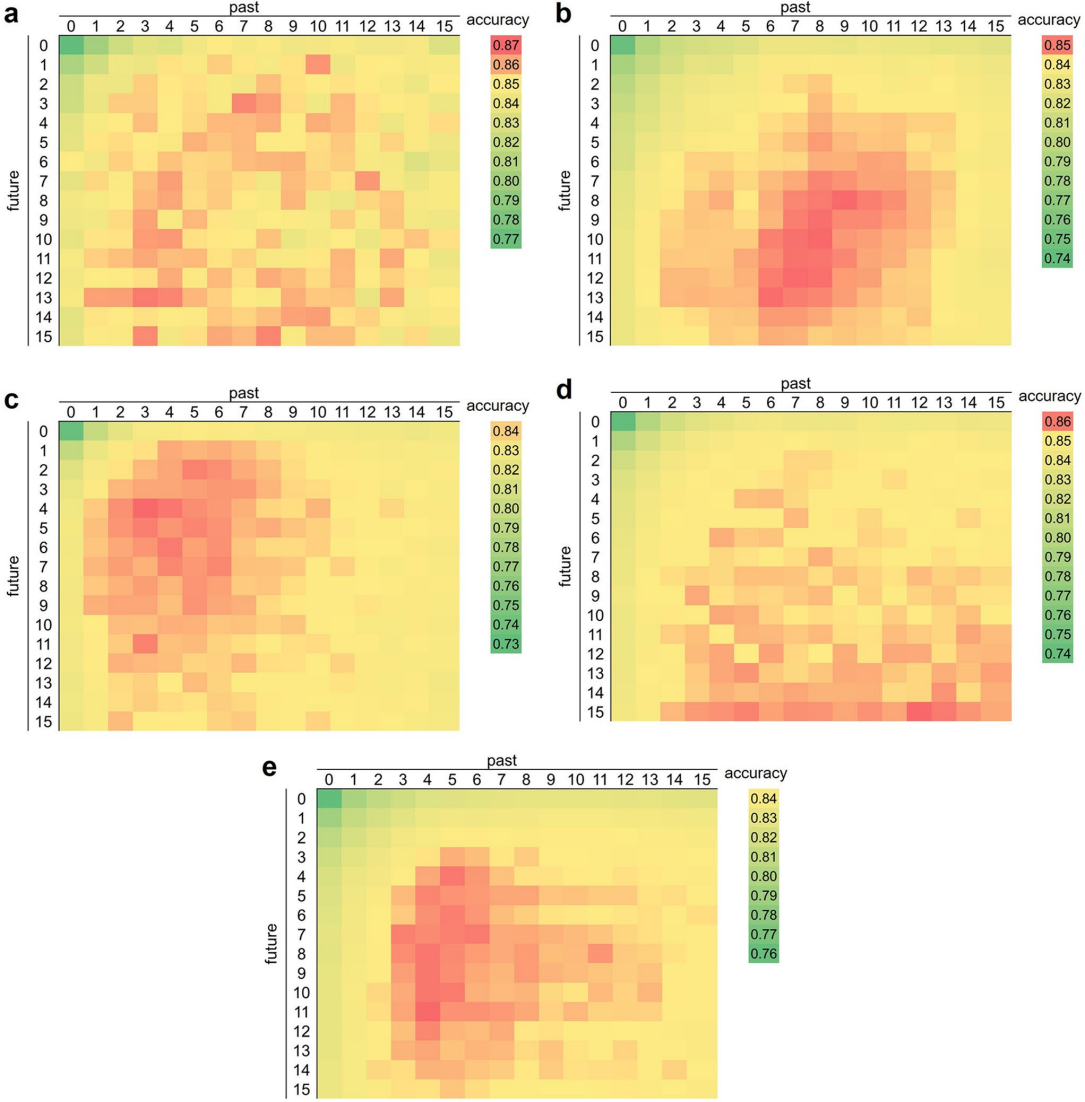
Five-fold cross-validation accuracy for different time window combinations on the released set. The accuracy for each time window combination is indicated by a gradient from green to red. The vertical axis indicates the future time window size, and the horizontal axis indicates the past time window size (minute). The time window size equal to 0 min represents the non-time window. (**a**) MLP, (**b**) SVM, (**c**) RF, (**d**) XGBoost, and (**e**) LR

The accuracy was maximum at (future, past) = (13, 3), (10, 8), (4, 3), (15, 12), and (11, 4) for the MLP (Fig. 3a), SVM (Fig. 3b), RF (Fig. 3c), XGBoost (Fig. 3d), and LR (Fig. 3e), respectively. The points with higher accuracy (red points in Fig. 3a) were unevenly distributed in the MLP. The accuracy around the point where the accuracy reached its highest value was higher than that of the other points in the SVM, RF, and LR. The accuracy at approximately (future) = 15 was higher than that at other points in XGBoost. Similar to all classifiers, the accuracy in the upper-left corner was lower than that at the other points (Fig. 3).

Pairs of past and future time-window sizes that maximized the accuracy of the grid search were applied to each classifier to examine how much the other indicators would improve. Thirty-five people were trained on the released set, and 35 people were validated for the withheld set. For all classifiers, the ACC, SEN, SPE, PRE, F1-score, and AUC were improved using both past and future data (Tables 1 and 2).

**Table 1:** SA detection performance (per-segment performance) comparison of models with different non-time window machine learning classifiers

Method	ACC (%)	SEN (%)	SPE (%)	PRE (%)	F1-score (%)	AUC
MLP	81.4	74.3	85.7	76.2	75.2	0.885
SVM	79.8	72.0	84.6	74.2	73.1	0.853
RF	79.4	68.8	85.9	75.0	71.8	0.867
XGBoost	79.5	70.4	85.1	74.4	72.4	0.869
LR	81.6	72.0	87.5	77.9	74.8	0.880

ACC: accuracy; SEN: sensitivity; SPE: specificity; PRE: precision; AUC: area under the receiver operating characteristic curve; MLP: multilayer perceptron; SVM: support vector machine; RF: random forest; XGBoost: extreme gradient boosting; LR: logistic regression

**Table 2:** SA detection performance (per-segment performance) comparison of models with different time window machine learning methods

Method	ACC (%)	SEN (%)	SPE (%)	PRE (%)	F1-score (%)	AUC
MLP	88.1	84.6	90.3	84.6	84.6	0.942
SVM	86.0	80.3	89.6	83.1	81.7	0.928
RF	87.1	79.1	92.1	86.2	82.5	0.943
XGBoost	88.6	85.0	90.9	85.7	85.4	0.952
LR	87.3	84.1	89.2	83.1	83.6	0.942

Future and past information were used. The time window size was determined for each classifier based on the grid search results

### Effect of time window size for per-recording SAS detection

**Fig. 4:**
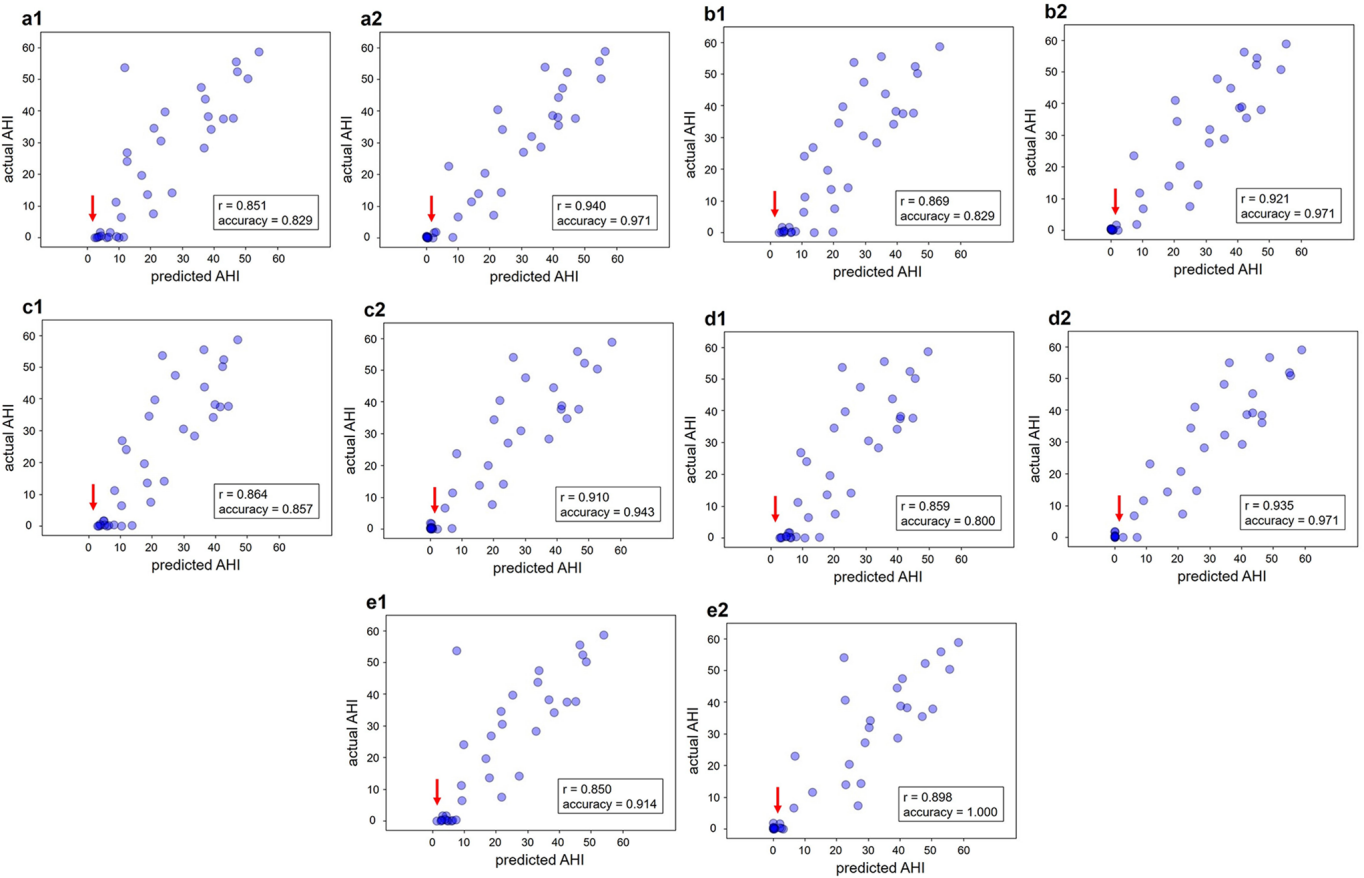
Correlation between predicted AHI and actual AHI. The classifiers were (**a**) MLP, (**b**) SVM, (**c**) RF, (**d**) XGBoost, and (**e**) LR. (a1, b1, c1, d1, e1) Non-time window (a2, b2, c2, d2, e2) Future and past information was used. Pearson’s correlation coefficient (indicated by r) and accuracy are shown in the box. The red arrows indicate areas where significant improvement was achieved by expanding the time window. The time window size was determined for each classifier from the results of the grid search

The ACC, SEN, SPE, PRE, F1-score, and correlation in SAS diagnosis were improved using both past and future data for all classifiers (Tables 3 and 4). The sensitivity reached 95.7–100%, and the specificity reached 91.7–100%, which is the estimation accuracy of the SAS. In particular, the specificity increased considerably. Regarding the AHI correlation diagram, healthy individuals who were incorrectly diagnosed with AHI ≥ 5 (SAS) in the non-time window were correctly classified as having AHI < 5 (normal) when the time window was expanded (Fig. 4). The spread of the lower-left point in the correlation diagram along the x-axis narrowed when the time window was expanded. Regarding the comparison of the classifiers, LR diagnosed SAS with the highest accuracy but with the lowest correlation coefficient.

**Table 3:** SAS diagnosis performance (per-recording performance) comparison of models with different non-time window machine learning methods

Method	ACC (%)	SEN (%)	SPE (%)	PRE (%)	F1-score (%)	Corr.
MLP	82.9	100.0	50.0	79.3	88.5	0.851
SVM	82.9	100.0	50.0	79.3	88.5	0.869
RF	85.7	100.0	58.3	82.1	90.2	0.864
XGBoost	80.0	100.0	41.7	76.7	86.8	0.859
LR	91.4	100.0	75.0	88.5	93.9	0.850

Corr: Pearson’s correlation coefficient

**Table 4:** SAS diagnosis performance (per-recording performance) comparison of models with different time window machine learning methods

Method	ACC (%)	SEN (%)	SPE (%)	PRE (%)	F1-score (%)	Corr.
MLP	97.1	100.0	91.7	95.8	97.9	0.940
SVM	97.1	100.0	91.7	95.8	97.9	0.921
RF	94.3	95.7	91.7	95.7	95.7	0.910
XGBoost	97.1	100.0	91.7	95.8	97.9	0.935
LR	100.0	100.0	100.0	100.0	100.0	0.898

Future and past information were used. The time window size was determined for each classifier based on the grid search results

## Discussion

This study primarily aimed to evaluate the usefulness of both past and future ECG data for detecting SA. The accuracy of detecting SA and diagnosing SAS was improved using both past and future ECG data, regardless of the machine learning classifier used. Past and future ECG information from the point of SA occurrence is generally effective in detecting SA. The detection accuracy decreased when the range of past and future data was narrow (0–2 min) or wide in the grid search for the range of data used. Thus, the data should be adjusted within the range of a few minutes. This reflects that SA is not a phenomenon that occurs in isolation but is continuous. The finding that future ECGs are effective in detecting SA also reflects that the heart rate decreases during SA but increases after SA [12]. In other words, the increase in the heart rate induced by SA affected the ECG waveform after the onset of SA.

A time window longer than one minute might inadvertently include unrelated SA events, potentially affecting detection accuracy. In this study, the width of target window was fixed at 1 min, while data before and after this window was used for predicting whether the target window represented SA. To identify the optimal time window, we extended it up to 15 min in both directions, which was considered sufficiently long. Previous studies suggest that one SA event may trigger another shortly after [24, 25]. Consequently, including earlier SA events within the time window could enhance detection accuracy by indicating the likelihood of subsequent events. A recent study using single-lead ECG data 120 s prior to predict SA has demonstrated substantially higher prediction accuracy when both 120-second prior period and target epoch included SA, compared with when only the target epoch included SA [26]. Based on these considerations, we conclude that including non-target SA events within the time window is unlikely to significantly reduce detection accuracy and may improve it.

When defining SAS as AHI ≥ 5, we achieved sensitivity ranging from 95.7 to 100% and specificity ranging from 91.7 to 100% as the estimation accuracy of SAS. A substantially higher accuracy was observed compared to a method using periodic heart rate variability (CVHR) obtained from the same single-channel ECG data, which achieved a sensitivity of 82% and specificity of 95% [27]. Furthermore, one of the sleep evaluation devices (controlled medical devices), “WatchPAT200” by Itamar Medical, showed a sensitivity of 95.8% and a specificity of 100% with an AHI threshold of five [28]. Our results are consistent with those of the previous study. Our results showed satisfactory accuracy of the SAS screening tests. If the aforementioned predictive results of machine learning are included in the report on a clinical Holter ECG test, further evaluation can be recommended with high confidence. This means that one can screen patients suspected of SAS from those who wear a Holter ECG owing to the suspicion of cardiovascular disease.

When interpreting the diagnostic accuracy, attention should be paid to the AHI range. LR had the highest diagnostic accuracy for SAS when AHI ≥ 5 was considered SAS but had the lowest correlation coefficient between the predicted AHI and the actual AHI (Tables 3 and 4). This means that the probability of misdiagnosing a healthy individual with an AHI < 5 as having an AHI of ≥ 5 (SAS) is low; however, the discrepancy between the predicted AHI and the actual AHI is large. In clinical practice, AHI ≥ 5 is the standard threshold for diagnosing SAS [23]. However, for classification based on severity, the AHI criteria should be 5 (mild), 15 (moderate), or 30 (severe), and the decision to initiate treatment is made for cases with moderate to severe AHI. A method for predicting the AHI that demonstrates a high correlation within specific ranges for purposes such as screening, diagnosis, and treatment, as well as across a broad range of AHI values, should be developed.

Regarding the differences among machine-learning classifiers, the results of this study showed that the optimal range of data used was different for each classifier. This may be owing to differences in their characteristics. MLP is a neural network, SVM is a kernel method, RF and XGBoost are decision trees, and LR is a multivariate analytical method. The differences between these methods may have affected the differences in the optimal data usage range. Among the five classifiers used in this study, SVM, RF, and LR exhibited unimodal accuracy, making them easier to use because they simplify the determination of the optimal time window.

SVM and RF emerge as the optimal choices among the five evaluated machine learning classifiers. This conclusion is based on several factors. SVM, RF, and LR exhibited unimodal accuracy patterns, simplifying time window selection and enhancing their model-based predictive usability. Among these three classifiers, LR demonstrated a slightly lower correlation coefficient for SAS diagnosis (AHI ≥ 5) compared with the others when time windows were applied. This suggests that LR’s accuracy might decrease more significantly than might the other classifiers if SAS diagnostic criteria were to change. Furthermore, LR achieved 100.0% in all metrics calculated in this study except for the correlation coefficient, raising concerns about potential overfitting. Using time windows, RF performed better in terms of SA detection, while SVM excelled in SAS diagnosis (AHI ≥ 5). Additionally, RF generally demonstrates faster computation than does SVM. The final selection between SVM and RF might depend on the specific application and available computational resources. Both classifiers showed strengths that made them preferable to the other tested classifiers in this context.

Most SA events last between 10 and 30 s [29], suggesting that multiple SA events could occur within a single one-minute epoch. This possibility might affect both AHI count and subsequent diagnoses based on it. A previous study investigated the impact of epoch length on SA detection accuracy using ECG data [30]. They found that accuracy was higher during 60-second and 75-second epochs, whereas 15-second epochs yielded the lowest accuracy. This indicates that shorter epochs may lead to decreased SA detection accuracy. Considering these findings, reducing epoch length could negatively impact SA detection accuracy, consequently affecting AHI prediction accuracy. Conversely, longer epochs may improve SA detection accuracy; however, they might particularly pose a risk when multiple SA events occur within a single epoch, potentially influencing AHI prediction accuracy. Although our results in the current study demonstrated sufficiently high accuracy, we suggest that there is still room for improvement, taking the aforementioned factors into account. We have already initiated a separate foundational study to investigate the impact of epoch length on prediction accuracy. Future predictive models are warranted for incorporating these considerations to further enhance accuracy.

This study has some limitations. First, we used a small dataset of 70 individuals, which is insufficient to generalize our results. Second, the dataset did not include patients with disorders other than sleep apnea [12]. In future research, increasing the amount of data and including patients with more complex apneic cases, such as insomnia, is necessary to verify whether the results can be generalized. Therefore, we are currently working at our university hospital to collect more patient data and expand our dataset.

## Conclusions

We employed machine learning techniques to estimate the occurrence of SA during sleep using 70 single-channel ECG recordings. The predictive accuracy of SA was improved by utilizing data from both past and future SA occurrences. This improvement was observed in all the machine learning classifiers (MLP, SVM, RF, XGBoost, and LR), whereas the optimal range of the time window varied among the classifiers. Moreover, when diagnosing SAS with AHI ≥ 5, the diagnostic accuracy improved compared to using medical devices when incorporating data from the past to the future. Our results suggest that it is possible to conduct screening tests for SAS with clinical-level accuracy by using data obtained from single-lead ECGs with appropriate past and future time windows.

## Data Availability

The experimental data that support the findings of this study are available in PhysioNet with the identifier https://physionet.org/content/apnea-ecg/1.0.0/. The other data presented in this study are available by reasonable request through the corresponding author.
